# A Singularly Painful Affection of the Appendages of the Left Eye, from an Uncommon Cause, Successfully Treated

**Published:** 1816-12

**Authors:** John Stevenson

**Affiliations:** Member of the Royal College of Surgeons, and Lecturer on the Eye, and Ear, &c. &c.


*For the London Medical and Physical Journal.*

*A singularly painful Affection of the Appendages of the left Eye, from an uncommon Cause, successfully treated.* By JOHN STEVENSON, Esq. Member of the Royal College of Surgeons, and Lecturer on the Eye, and Ear, &c. &c.

ON the 20th of May 1815, Miss H. Cole, seven years of age, of a remarkably healthy constitution, without any known cause, was seized with a pain in the forehead directly over the left eye, which gradually increased until it became almost insupportable. Having, in a few minutes, attained its acme, it as suddenly subsided, leaving her in every respect as well as before the attack commenced. After some hours interval, the paroxysm returned in a similar manner, the disease assuming a perfectly remittent character, recurring at irregular periods several times a day; its duration was from a few minutes to a quarter of an hour. Those fits which took place during the afternoon and evening, were more particularly urgent and severe. Occasionally she awoke with the agonizing pain, which, after lasting for a very short time, wholly left her, when she generally fell again into a quiet repose. After thus partially affecting the forehead for the space of a week, the seat of the complaint was transferred to the corresponding palpebræ, the pain, which was equally sudden and intense, commencing at the inner, and extending to the outer canthus, in the direction of the fibres of the orbicularis muscle. There was not the slightest uneasiness in the ball of the eye, any redness, or even fulness of the vessels of the conjunctiva, or increased secretion of tears; nor were the integuments of the eyelids in any degree discoloured.

By the suddenness with which the pain came on, its extreme severity whilst it continued, and its abrupt termination, it bore a striking resemblance with tic douloureux. The only accompanying constitutional derangement was an affection of the stomach, characterised by a sort of convulsive regurgitation of the food she had recently taken, by mouthfuls, without experiencing at the time any sensation of sickness, or effort to vomit. The substances rejected consisted of little more than the undigested aliment she had last swallowed, unmixed with vitiated secretion, or diseased bile.

She had taken, for about three weeks, under the direction of her apothecary, a draught of Magnes. Sulphat. in Infus. Rosæ ter. de die, by which the bowels were preserved in a perfectly open state; a blister had been likewise applied to the the temple, another behind the ear of the affected side, and a fomentation of poppy-heads to the part in pain.

These means not affording relief, she was recommended by her noble patroness, the Countess of L——, to my care.

Knowing, from experience, that many anomalous complaints of the organ of vision arise from sympathy with the *primæ viæ*, a due consideration of all the circumstances of this extraordinary case led me to suspect, that it probably derived its origin from that source. With this impression on my mind, I directed an emetic to be exhibited in the evening, and a powerful dose of Hydrarg. Submur. and Jalap, the following morning, requesting a strict examination of the respective discharges from the stomach, and alimentary canal. In order to appease the irritability of the stomach, after the cathartic had produced its full effect, a saline draught with Tinct. Opii was ordered to be taken *durante effervescentia* every four hours.

Regarding the pain in the eye-lid as purely sympathetic, I forbore prescribing any kind of topical application, at least, until the nature of the disease should be more clearly developed, or its dependance upon gastric or intestinal irritation ascertained. Although, by the action of the emetic, nothing of an offensive nature was thrown up from the stomach, yet, after its operation, my little patient felt not the slightest return of the accustomed pain. Five copious slimy and greenish fœcal evacuations were the result of the purgative. In the last of these was discovered the *exciting cause* of this distressing malady: viz. *a large coral bead.* She now recollected that on untying, by the aid of her teeth, a necklace belonging to her lady, the thread had broken in her mouth, which became, in consequence, filled with the disengaged beads, one of which must have been swallowed.

After this, the symptoms wholly subsided, and nothing was required excepting a little medicine to restore the appetite, and keep the bowels regular.

The above case is entitled to attention in a practical view, on account of the singularly anomalous character of the symptoms, the detection of their exciting cause, and their successful removal. In the course of my practice exclusively in the treatment of diseases of the eye and ear, I have more than once met with instances of verminous, and other kinds of intestinal irritation, producing sometimes ptosis of one, or both of the superior palpebræ, and occasionally spasmodic affection of the muscles, and consequent distortion of the globe of the eye. But, in none of these cases did any severe remittent pains prevail, as in the one under consideration.

 This

This statement affords also an additional illustration to those already on record, of the not very uncommon, though not less important pathological fact, relative to the close sympathy which is now and then proved to subsist between the eye and its appendages, and the *primæ viæ;* a circumstance never to be lost sight of in the management of ocular ailments. It points out likewise the great benefit that may be rendered to the suffering patient, and the credit that may be gained by the practitioner, from his knowledge of the laws of sympathy,—a subject so ably treated of by the late Mr. Hunter. This is a study which I would beg leave, in an especial manner, to recommend to those who are just entering upon their medical career. Without experiencing how greatly the visual organ depends for many of its morbid phenomena upon visceral irritation for their primary cause, I might have persisted in applying remedies to a part which, though apparently the true seat of the complaint, there was good reason to believe suffered only secondarily by sympathy. Add to this,—had the symptoms been allowed to remain long protracted, besides the exquisite sufferings of the patient by their continuance, they might have ushered in others of a description still more alarming, and eventually terminated in the most disastrous and even fatal consequences.

I will just suggest, by way of conclusion, that from the irritable condition of the stomach, and the cessation of the pain immediately after the operation of the emetic, it is not unlikely that the extraneous substance which was found to occasion the disease, had actually lodged in that viscus, and that it was protruded through its pyloric extremity into the duodenum by the effort of vomiting, and finally expelled from the alimentary tube, by the powerful and efficient drastic cathartic.

*Great Russell-street, Bloomsbury;*
*November* 19, 1816.

